# Metabolic profiling identifies the significance of caffeine metabolism in CKD

**DOI:** 10.3389/fbioe.2023.1006246

**Published:** 2023-02-17

**Authors:** Xinghua Guo, Hongquan Peng, Peijia Liu, Leile Tang, Jia Fang, Chiwa Aoieong, Tou Tou, Tsungyang Tsai, Xun Liu

**Affiliations:** ^1^ Department of Rheumatology, The Third Affiliated Hospital of Sun Yat-Sen University, Guangzhou, Guangdong, China; ^2^ Department of Nephrology, Kiang Wu Hospital, Macau, Macao SAR, China; ^3^ Department of Nephrology, GuangZhou Eighth People’s Hospital, GuangZhou Medical University, Guangzhou, Guangdong, China; ^4^ Department of Cardiovasology, The Third Affiliated Hospital of Sun Yat-Sen University, Guangzhou, Guangdong, China; ^5^ Department of Nephrology, The Third Affiliated Hospital of Sun Yat-Sen University, Guangzhou, Guangdong, China

**Keywords:** metabolomics, chronic kidney disease, glomerular filtration rate, caffeine metabolism, caffeine

## Abstract

**Background:** With the development of chronic kidney disease (CKD), there are various changes in metabolites. However, the effect of these metabolites on the etiology, progression and prognosis of CKD remains unclear.

**Objective:** We aimed to identify significant metabolic pathways in CKD progression by screening metabolites through metabolic profiling, thus identifying potential targets for CKD treatment.

**Methods:** Clinical data were collected from 145 CKD participants. GFR (mGFR) was measured by the iohexol method and participants were divided into four groups according to their mGFR. Untargeted metabolomics analysis was performed *via* UPLC-MS/MSUPLC–MSMS/MS assays. Metabolomic data were analyzed by MetaboAnalyst 5.0, one-way ANOVA, principal component analysis (PCA), and partial least squares discriminant analysis (PLS-DA) to identify differential metabolites for further analysis. The open database sources of MBRole2.0, including KEGG and HMDB, were used to identify significant metabolic pathways in CKD progression.

**Results:** Four metabolic pathways were classified as important in CKD progression, among which the most significant was caffeine metabolism. A total of 12 differential metabolites were enriched in caffeine metabolism, four of which decreased with the deterioration of the CKD stage, and two of which increased with the deterioration of the CKD stage. Of the four decreased metabolites, the most important was caffeine.

**Conclusion:** Caffeine metabolism appears to be the most important pathway in the progression of CKD as identified by metabolic profiling. Caffeine is the most important metabolite that decreases with the deterioration of the CKD stage.

## 1 Introduction

Chronic kidney disease (CKD) is a chronic disorder involving structural and functional organ changes. There are multiple underlying causes of this disorder which is characterized by its irreversibility and progressive development. CKD is a growing public health concern worldwide. As the disease progresses, the kidney’s ability to remove nitrogenous wastes, exogenous molecules, and metabolism of low molecular weight proteins decrease, resulting in multiple clinical sequelae. It is recognized that metabolites change with the development of kidney injury (Rysz et al., 2021). However, the effect of these metabolites on the etiology, progression, and prognosis of CKD remains unclear. The main clinical challenge is that current medical interventions aimed at delaying kidney decline are very limited. As such, the discovery of potential metabolic pathways could help us identify new therapeutic targets and address this serious problem (Chen et al., 2019a).

Several attempts have been made to identify new CKD-associated biomarkers and deepen our understanding of the pathological mechanisms underlying CKD (Dou et al., 2018; Xu et al., 2018). Metabolomics can be used to uncover metabolites associated with critical kidney functions such as the glomerular filtration rate (GFR), allowing for important clinical information to be obtained from biological samples (Earl et al., 2018; Lai et al., 2018) Rhee *et al.* reported that ten plasma metabolites are nominally associated with CKD progression (Rhee et al., 2016). In addition, Chen *et al.* found that five metabolites, including 5-methoxytryptophan (5-MTP), canavaninosuccinate (CSA), acetylcarnitine, tiglylcarnitine, and taurine, can be used to accurately identify the clinical stages of CKD, and that tryptophan hydroxylase-1 (TPH-1) presents a potential therapeutic target in CKD (Chen et al., 2019b). Feng *et al.* found that CKD rats can be differentiated from sham rats by metabolites involved in the pathways of gut microbial metabolism. They also found that improvement of gut dysbiosis retarded the progression of kidney disease in a rat model of CKD (Feng et al., 2019). Zhao *et al.* utilized an adenine-induced CKD model to identify perturbations in fatty acid metabolism, purine metabolism, and amino acid metabolism (Zhao et al., 2014). Brunetto *et al.* compared the serum metabolic profile of healthy and CKD dogs and found decreased urea, creatinine, creatine, citrate, lipids, lactate, branched-chain amino acids (BCAAs), and glutamine in CKD; a specific diet was able to maintain and retard the progression of CKD (Brunetto et al., 2021). Lanzon *et al.* analyzed the serum and urine of patients with severe obesity and CKD before and after undergoing bariatric surgery (BS) and found that isoleucine and tyrosine were increased in CKD patients compared to those without CKD (Lanzon et al., 2021). Gordin *et al.* (Gordin et al., 2019) used metabolic pathway analysis and reported that hexose, mitochondrial, amino acid, and purine pathways are associated with preserved kidney function.

Factors found to accelerate kidney decline have been explored in many studies. Most research involving metabolic profiling has focused on metabolites that increase with deteriorating renal function. However, in this study, we have focused on identifying the metabolic pathways associated with improved kidney function. By utilizing metabolic profiling, we attempted to identify metabolites that decrease as CKD deteriorates, ascertain the most relevant metabolic pathways, and identify potential therapeutic targets to improve kidney function.

## 2 Materials and methods

### 2.1 Participants and mGFR measurement

All participants provided informed consent before participating in the study according to a protocol approved by the Kiang Wu Hospitalethics committee. All participants were recruited in August 2019 from the Kiang Wu Hospital (Santo Antonio, Macau) and *via* outpatient clinics. The cohort included 145 patients who met the study inclusion criteria and were diagnosed with CKD based on the NKF-KDOI guidelines. Peripheral venous blood (4 mL) was collected from each patient; plasma samples were used for metabolomic analysis. Renal function was evaluated using GFR (mGFR) by utilizing the plasma clearance of iohexol (Shah et al., 2013). After blood was collected for the aforementioned tests, Iohexol was injected over 2 min (300 mg/mL, GE Healthcare, Shanghai, China) and plasma (6 mL) was collected from the contralateral upper extremity to detect the Iohexol concentration (by HPLC) at 120 and 240 min after Iohexol administration. For participants with eGFR<30 min/mL/1.73 m (Chen et al., 2019a), the blood collection times were changed to 120 and 300 min. All blood samples were centrifuged at 2000 *g* for 10 min at room temperature to extract plasma and stored at −80°C until analysis.

### 2.2 UPLC–MS/MS assays

All of the untargeted metabolomics analyses were conducted at the Dian Calibra-Metabolon Joint Metabolomics Laboratory (Hangzhou, China). Four different UPLC–MS/MS assays of small molecule metabolites were performed on each sample (Shen et al., 2020). Automatic liquid transfer during sample preparation was handled on a Hamilton automated MicroLab STAR^®^ system (Hamilton, Switzerland). A methanol-based sample extraction solution was added to each sample and mixed using a GeneGrinder 2010 (Spex SamplePrep, United States of America) mixer. After 2 minutes of vigorous shaking and centrifugation to precipitate proteins and other debris, the extracted metabolites in the supernatant were collected and divided into four fractions: two fractions were analyzed by reversed-phase (RP) UPLC-MS/MS under positive electrospray ionization (ESI) mode. The two UPLC methods were slightly different using the same column (BEH C18 2.1 × 100 mm, 1.7 μm column, Waters). The mobile solutions for the two positive ESI UPLC-MS/MS were water and methanol containing 0.05% perfluoropentanoic acid (PFPA) and 0.1% formic acid (FA). The third fraction was used for reversed-phase UPLC-MS/MS in negative ion ESI mode (BEH C18 2.1 × 100 mm, 1.7 μm column, Waters), and the mobile solutions were methanol and water in 6.5 mM ammonium bicarbonate at pH 8. The last fraction was used for hydrophilic interaction liquid chromatography (HILIC)/UPLC-MS/MS in negative ESI mode (BEH Amide 2.1 × 150 mm, 1.7 μm column, Waters), and the mobile solutions consisted of water and acetonitrile with 10 mM ammonium formate at pH 10.8. Each fraction was dried under nitrogen gas flow and then dissolved in reconstitution solutions before being injected into each of the four UPLC-MS/MS systems. The QE mass spectrometer was alternated between full MS and data-dependent MS2 scans using dynamic exclusion for data collection. The scan range was 70–1,000 m/z. Processing, extraction, and peak identification of the raw mass spectrometry data were carried out using in-house developed software, and metabolites were identified by comparing the experimental ion characteristics to entries in an in-house library which was constructed using pure reference standards. The entries in the library included retention time/retention index (RI), mass to charge ratio (m/z), and MS/MS spectral data of each reference standard.

### 2.3 Metabolomics statistical analysis

All participants were divided into four groups according to mGFR: group A (mGFR<30 mL/min/1.73 m (Chen et al., 2019a)), group B (30 mL/min/1.73 m (Chen et al., 2019a)≤mGFR<60 mL/min/1.73 m (Chen et al., 2019a)), group C (60 mL/min/1.73 m (Chen et al., 2019a)≤mGFR <90 mL/min/1.73 m (Chen et al., 2019a)), and group D (mGFR ≥90 mL/min/1.73 m (Chen et al., 2019a)). Statistical analysis of patient data was carried out using SPSS 26.0. Statistical significance was determined using a threshold of *p* = 0.05. Metabolomic data analyses were carried out using MetaboAnalyst 5.0 (https://www.metaboanalyst.ca). The mass spectrometry data which were acquired by untargeted metabolomics analysis were uploaded as comma separated values (.csv). The uploaded data file contains a data matrix of 145 (samples) × 1,094 (compounds). Before data analysis, a data integrity check was performed to ensure that all the necessary information had been collected. To minimize bias associated with the omission of censored data, all missing and zero values were replaced by half of the minimum positive values across samples in the original data. Normalization was done *via* log transformation and Pareto scaling.

One-way analysis of variance (ANOVA), principal component analysis (PCA), and partial least squares-discriminant analysis (PLS-DA) were used to screen out the differential metabolites. MetaboAnalyst 5.0 provided one-way ANOVA test results to determine whether the overall comparison among each group was significant. Univariate analyses provided a preliminary overview of features that are potentially significant in discriminating the conditions under investigation. Statistical significance was determined using a threshold of *p* = 0.05.

PCA and PLS-DA were also performed using MetaboAnalyst 5.0. PCA is an unsupervised method aiming to find the directions that best explain the variance in a data set X) without referring to class labels Y). The data are summarized into much fewer variables called scores, which are weighted averages of the original variables. PLS is a supervised method that uses multivariate regression techniques to extract, *via* a linear combination of the original variables X), information that can predict class membership Y). To assess the significance of class discrimination, a permutation test was performed. In each permutation, a PLS-DA model was built between the data X) and the permuted class labels Y) using the optimal number of components determined by cross-validation for the model based on the original class assignment. MetaboAnalyst supports two types of test statistics for measuring class discrimination. The first one is based on prediction accuracy during training. The second is the separation distance based on the ratio of the between-group sum of the squares and the within-group sum of squares (B/Wratio). If the observed test statistic is part of the distribution based on the permuted class assignments, the class discrimination cannot be considered statistically significant. Variable Importance in Projection (VIP), which is an important variable in PLS-DA, is a weighted sum of squares of the PLS loadings taking into account the amount of explained Y-variation in each dimension. VIP scores are calculated for each component. When more components are used to calculate the feature importance, the average of the VIP scores is used. A VIP threshold >1.0 was considered statistically significant.

### 2.4 Pathway analysis

Metabolic pathways were identified by utilizing open database sources of MBRole2.0 (http://csbg.cnb.csic.es/mbrole2/), including KEGG and HMDB. Compound names of the differential metabolites were first converted to KEGG IDs using MetaboAnalyst 5.0 and the KEGG IDs were submitted to MBRole 2.0 for KEGG pathway analysis.

## 3 Results

### 3.1 Characteristics of the study populations

We recruited 145 individuals aged 20 to 96 years, 68 of whom were male. Based on mGFR, 22 were assigned to group A, 47 to group B, 39 to group C, and 37 to group D. Table 1 presents the summary statistics for each group. Age, weight, body mass index (BMI), systolic blood pressure, diastolic blood pressure, creatinine, eGFR, and mGFR were symmetrically distributed. Arithmetic means and standard deviations are provided. The *p*-values of one-way ANOVA tests are also presented. Creatinine, mGFR and eGFR were used a trend test, and the Ptrend values were presented. The height followed an asymmetric distribution, and thus median and interquartile ranges are shown. The *p*-values of the Kruskal–Wallis test are presented. Sex, current smoking, current drinking, diabetes, hypertension, coronary heart disease, stroke, hyperuricemia, use of antiplatelet drugs, antilipemic agents, antihypertensive agents, hypoglycemic agents, immunosuppressors, and uric acid reduction medicine were dichotomous variables. For these, quantities and frequencies are shown as appropriate. The *p*-values of Chi-square tests are presented. There were no cases of current drinking, stroke, and immunosuppressive drug use.

**TABLE 1:** Clinical characteristics of each group’s participants.

Variables	Group				*p*
	A	B	C	D	
Age (y)	74.9 ± 16.5	72.7 ± 13.5	60.6 ± 14.1	41.8 ± 10.6	<0.01
Sex (M/F)	9/13	25/22	18/21	16/21	0.66
Height (cm)	154.0 (151.0, 166.5)	159.0 (155.0, 167.0)	157.0 (153.0–172.0)	163.0 (157.0–171.0)	0.43
Weight (kg)	62.3 ± 11.2	62.5 ± 17.0	66.2 ± 15.1	64.6 ± 14.1	0.85
BMI (cm/kg^2)	25.1 ± 3.2	24.6 ± 5.7	25.2 ± 4.7	23.9 ± 4.0	0.64
Systolic blood pressure (mmHg)	130.9 ± 14.2	132.6 ± 16.9	133.0 ± 16.8	126.7 ± 12.8	0.27
Diastolic blood pressure (mmHg)	70.7 ± 13.7	72.8 ± 13.0	77.6 ± 12.1	77.5 ± 12.1	0.08
Creatinine (mg per 100 mL)	241.3 ± 144.2	105.8 ± 26.5	80.9 ± 18.0	70.2 ± 18.9	<0.01*
eGFR (ml/(min.1.73 m (Chen et al., 2019a)))	23.2 ± 11.5	48.8 ± 15.5	75.5 ± 12.0	103.1 ± 15.9	<0.01*
mGFR (ml/(min.1.73 m (Chen et al., 2019a)))	21.9 ± 6.4	44.6 ± 7.9	73.2 ± 8.4	106.9 ± 12.3	<0.01*
current smoking	0	0	0	1 (2.7%)	0.40
current drinking	0	0	0	0	-
diabetes	10 (45.5%)	11 (23.4%)	7 (17.9%)	4 (10.8%)	0.02
hypertension	15 (68.2%)	28 (59.6%)	15 (38.5%)	4 (10.8%)	<0.01
coronary heart disease	9 (40.9%)	11 (23.4%)	5 (12.8%)	2 (5.4%)	0.004
stroke	0	0	0	0	-
hyperuricemia	0	8 (17.0%)	11 (28.2%)	2 (5.4%)	<0.01
antiplatelet drugs	9 (40.9%)	11 (23.4%)	5 (12.8%)	2 (5.4%)	<0.01
antilipemic agent	2 (9.1%)	5 (10.6%)	6 (15.4%)	0	0.13
anti-hypertensive agent	15 (68.2%)	28 (59.6%)	15 (38.5%)	4 (10.8%)	<0.01
hypoglycemic agent	10 (45.5%)	11 (23.4%)	7 (17.9%)	4 (10.8%)	0.02
immunosuppressor	0	0	0	0	-
Uric acid reduction medicine	0	8 (17.0%)	11 (28.2%)	2 (5.4%)	<0.01

Group A:mGFR<30 mL/min/1.73m2); group B: 30 mL/min/1.73m2 ≤ mGFR<60 mL/min/1.73m2); group C:60 mL/min/1.73m2 ≤ mGFR <90 mL/min/1.73m2); group D:mGFR ≥90 mL/min/1.73m2).

* Ptrend value.

### 3.2 Univariate analysis

**FIGURE 1:**
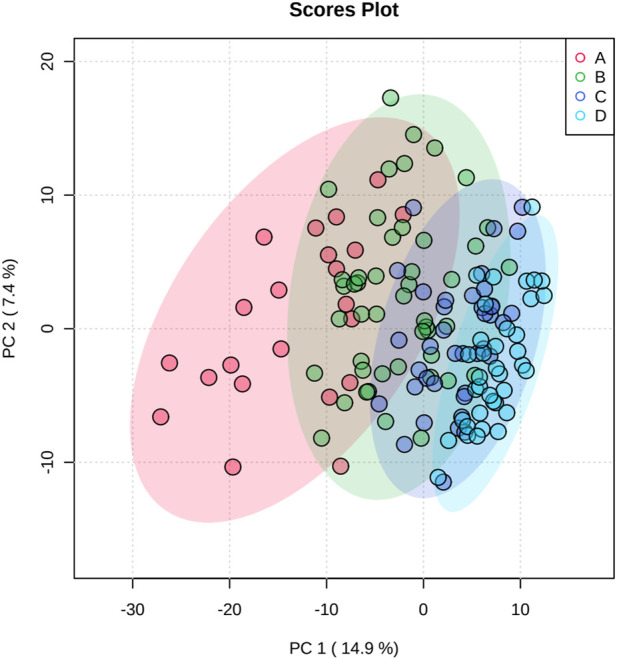
Scores plot between the selected PCs. The explained variances are shown in brackets.

Before data analysis, a data integrity check was performed to make sure that all the necessary information had been collected. The data normalization result implemented by MetaboAnalyst5.0 provided in Figure 1 supplement. After one-way ANOVA for multigroup analysis, Table 2 supplement shows all significant metabolites selected by ANOVA with *p*-value threshold 0.05 and Table 2 details these findings for the top 50 metabolites. ANOVA only tells whether the overall comparison is significant or not, it is followed by *post hoc* analyses in order to identify which two levels are different (Table 2 supplement).

**TABLE 2:** Top 50 features identified by one-way ANOVA.

	Compounds	f.value	p.value	-log10 (p)	FDR
1	erythronate	153.67	3.02E-44	43.52	3.21E-41
2	N-acetylneuraminate	141.72	2.27E-42	41.644	1.21E-39
3	hydroxyasparagine	136.69	1.51E-41	40.82	5.36E-39
4	C-glycosyltryptophan	130.7	1.56E-40	39.807	4.14E-38
5	gulonate	126.55	8.22E-40	39.085	1.75E-37
6	N-acetylserine	121.06	7.87E-39	38.104	1.39E-36
7	N-acetyltaurine	118.59	2.23E-38	37.652	2.92E-36
8	3-(3-amino-3-carboxypropyl)uridine	118.48	2.34E-38	37.631	2.92E-36
9	5-methylthioribose	118.35	2.47E-38	37.607	2.92E-36
10	N,N-dimethyl-pro-pro	101.13	5.63E-35	34.25	5.98E-33
11	N2,N2-dimethylguanosine	100.75	6.74E-35	34.172	6.50E-33
12	4-acetamidobutanoate	99.782	1.07E-34	33.971	9.46E-33
13	pseudouridine	97.579	3.09E-34	33.509	2.53E-32
14	N6-carbamoylthreonyladenosine	94.4	1.48E-33	32.831	1.12E-31
15	O-sulfo-L-tyrosine	84.812	2.05E-31	30.689	1.45E-29
16	arabonate/xylonate	84.29	2.70E-31	30.568	1.79E-29
17	N-acetylalanine	84.099	2.99E-31	30.524	1.87E-29
18	N-acetylhomocitrulline	83.616	3.88E-31	30.411	2.29E-29
19	gluconate	80.378	2.26E-30	29.646	1.26E-28
20	hydroxy-N6,N6,N6-trimethyllysine	80.288	2.38E-30	29.624	1.26E-28
21	N,N,N-trimethyl-alanylproline betaine (TMAP)	78.643	5.92E-30	29.227	3.00E-28
22	5,6-dihydrouridine	77.297	1.26E-29	28.899	6.09E-28
23	1-methylhistidine	76.377	2.13E-29	28.673	9.81E-28
24	N1-methylinosine	70.241	7.63E-28	27.118	3.38E-26
25	3-methylglutaconate	68.344	2.40E-27	26.62	1.02E-25
26	3-hydroxy-3-methylglutarate	67.572	3.84E-27	26.416	1.57E-25
27	N4-acetylcytidine	67.292	4.56E-27	26.341	1.79E-25
28	alpha-ketoglutaramate	67.205	4.81E-27	26.318	1.82E-25
29	sulfate	66.705	6.54E-27	26.184	2.39E-25
30	vanillylmandelate (VMA)	65.656	1.25E-26	25.902	4.44E-25
31	glucuronate	63.813	3.99E-26	25.4	1.37E-24
32	2,3-dihydroxy-5-methylthio-4-pentenoate (DMTPA)	63.459	4.99E-26	25.302	1.66E-24
33	acisoga	63.356	5.33E-26	25.274	1.71E-24
34	1-ribosyl-imidazoleacetate	63.156	6.05E-26	25.218	1.87E-24
35	mannonate	63.128	6.16E-26	25.211	1.87E-24
36	dimethylarginine (SDMA + ADMA)	63.007	6.65E-26	25.177	1.96E-24
37	N-formylmethionine	61.59	1.65E-25	24.782	4.74E-24
38	5-(galactosylhydroxy)-L-lysine	61.132	2.22E-25	24.654	6.21E-24
39	urea	59.762	5.43E-25	24.265	1.48E-23
40	4-hydroxyphenylacetylglutamine	59.206	7.83E-25	24.106	2.08E-23
41	3′-sialyllactose	57.834	1.95E-24	23.71	5.05E-23
42	2-O-methylascorbic acid	56.139	6.11E-24	23.214	1.54E-22
43	2-pyrrolidinone	56.02	6.62E-24	23.179	1.64E-22
44	maltose	55.803	7.68E-24	23.115	1.85E-22
45	myo-inositol	54.891	1.43E-23	22.843	3.38E-22
46	1-methylguanidine	54.085	2.50E-23	22.602	5.78E-22
47	quinolinate	53.58	3.55E-23	22.449	8.03E-22
48	3-methylglutarylcarnitine 2)	53.062	5.10E-23	22.292	1.13E-21
49	N6-succinyladenosine	51.995	1.08E-22	21.966	2.34E-21
50	creatinine	51.229	1.86E-22	21.73	3.96E-21

### 3.3 Principal component analysis (PCA)

Figure 1 shows the results of the PCA; the separation trend among each group is shown, indicating that each group had a unique metabolic spectrum.

### 3.4 Partial least squares-discriminant analysis (PLS-DA)

**FIGURE 2:**
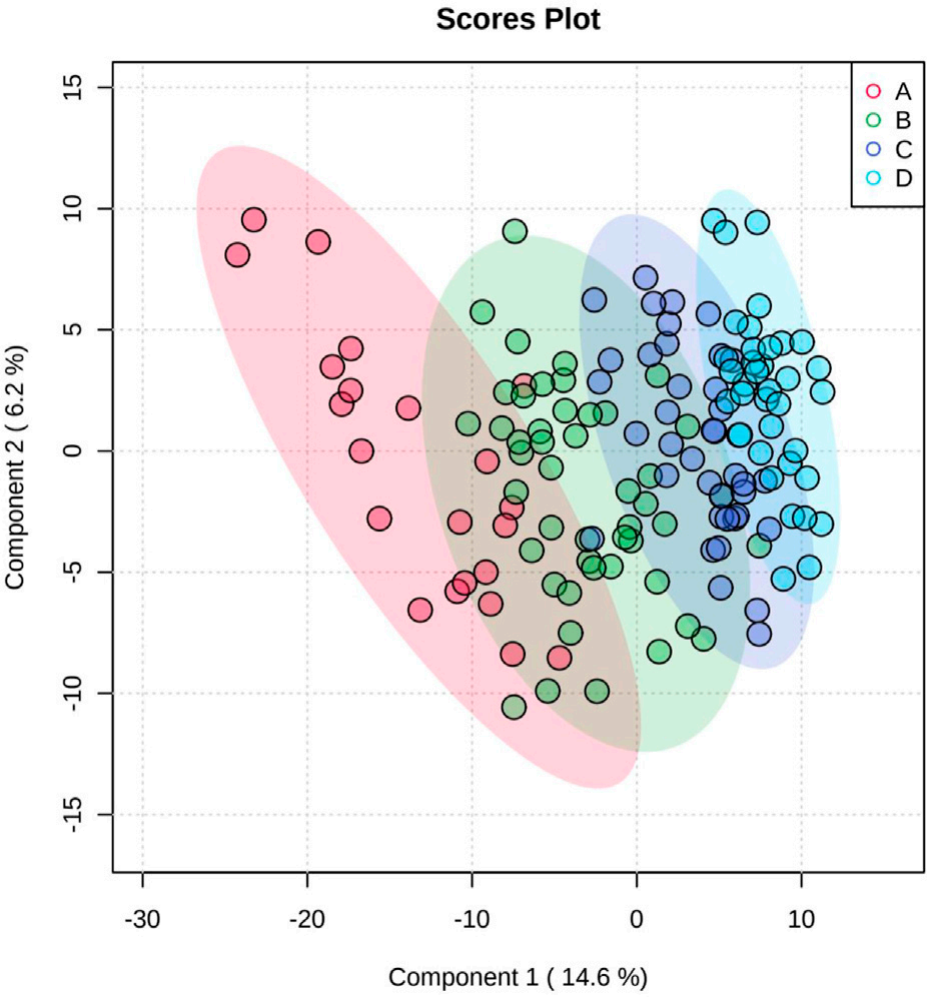
Scores plot between the selected PCs. The explained variances are shown in brackets.

**FIGURE 3:**
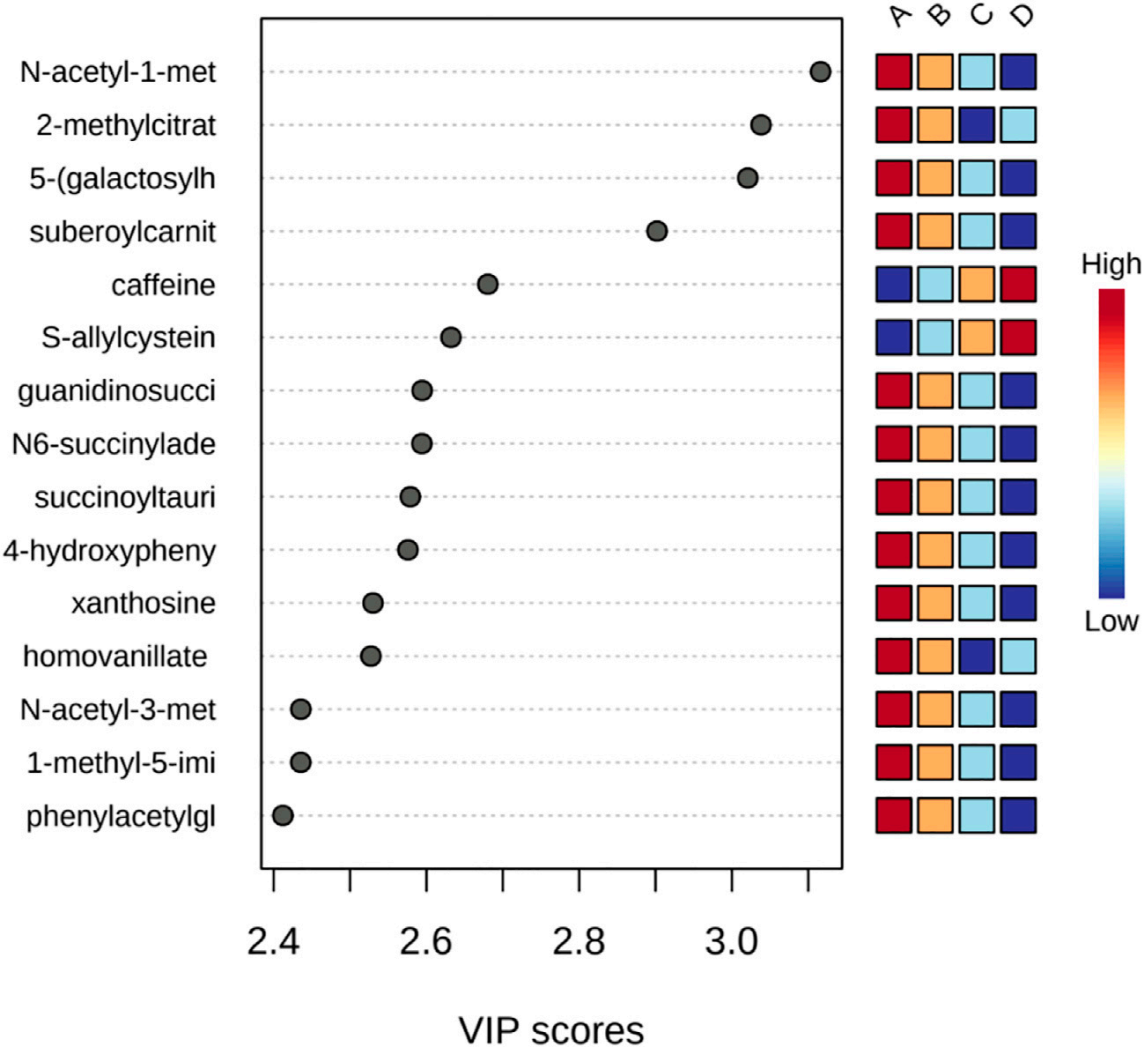
Important features identified by PLS-DA. The colored boxes on the right indicate the relative concentrations of the corresponding metabolite in each group under study.

PLS-DA was used to better distinguish the overall difference in the metabolic spectrum among each group and determine the metabolites that were most characteristic of each group. Figure 2 shows the 2D score plot between selected components; Figure 2 supplement shows the classification performance with different number of components; and Figure 3 shows important features identified by PLS-DA. To assess the significance of class discrimination, a permutation test was performed. In each permutation, a PLS-DA model was built between the data and the permuted class labels using the optimal number of components determined by cross validation for the model based on the original class assignment (Figure 3 supplement). VIP scores are calculated for each component. When more than components are used to calculate the feature importance, the average of the VIP scores was used. The average VIP value of caffeine was the highest among the metabolites that decreased with mGFR deterioration.

### 3.5 Pathway analysis

**FIGURE 4:**
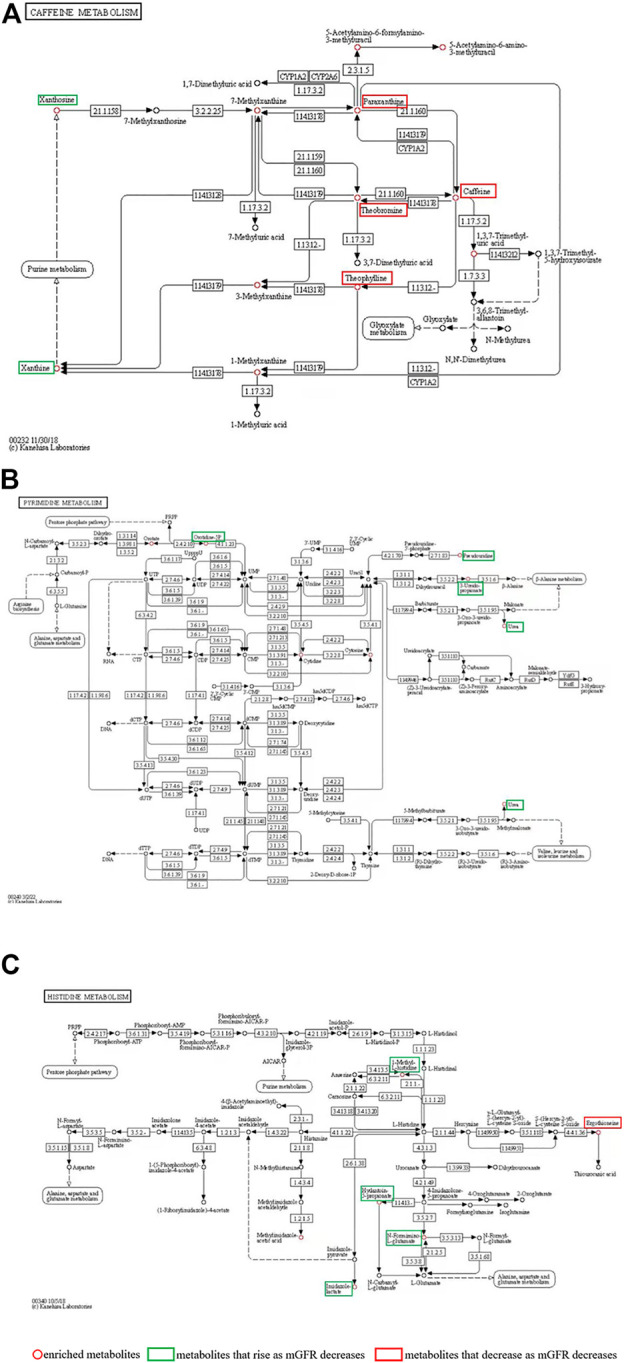
**(A)** Caffeine metabolism pathways; **(B)** Pyrimidine metabolism; **(C)** Histidine metabolism. The red circle represents the enriched metabolites; the green boxes are metabolites that rise as mGFR decreases; the red boxes are metabolites that decrease as mGFR decreases.

Differential metabolites were selected by one-way ANOVA, PCA, and PLS-DA. The differential metabolites were *p* < 0.05 in one one-way ANOVA and VIP>1 in PLS-DA. The metabolites of VIP>1 are presented in Table 3 plsda_vip supplement. The KEGG ID of each differential metabolite was found through MetaboAnalyst 5.0. KEGG IDs of differential metabolites were uploaded to MBRole2.0 and the background was set as the full database for KEGG pathway analysis. MBRole performs an overrepresented (enrichment) analysis of categorical annotations for a set of compounds of interest. These categorical annotations correspond to biological and chemical information available in several public databases and software. Caffeine metabolism, metabolic pathways, pyrimidine metabolism, and histidine metabolism were classified as important in Table 3. Caffeine metabolism was classified as the most important pathway compared with the three other identified pathways. Metabolic pathways included the other three metabolic pathways, so further analysis was directed at the metabolites enriched in these three metabolic pathways (Figure 4).

**TABLE 3:** Overview of metabolite set enrichment.

Annotation	Category	Set	In set	*p*-value	FDR correction
Cafffeine metabolism (map00232)	KEGG pathways	64	12	5.32e-18	2.98e-16
Metabolic pathway (map01100)	KEGG pathways	64	44	1.30e-9	3.64e-8
Pyrimidine metabolism (map00240)	KEGG pathways	64	8	1.22e-6	2.28e-5
Histidine metabolism (map00340)	KEGG pathways	64	6	2.81e-5	3.94e-4
Biosynthesis of alkaloids derived from histidine and purine (map01065)	KEGG pathways	64	5	1.09e-4	1.23e-3
Arginine and proline metobolism (map00330)	KEGG pathways	64	6	9.21e-4	8.59e-3
Phenylalanine metabolism (map00360)	KEGG pathways	64	4	3.17e-3	2.97e-2
Tryptophan metabolism (map00380)	KEGG pathways	64	5	5.26e-3	3.66e-2
ABC transporters (map00230)	KEGG pathways	64	5	8.20e-3	5.03e-2
Purine metabolism (map00230)	KEGG pathways	64	5	8.98e-3	5.03e-2
Ascorbate and aldarate metabolism (map00053)	KEGG pathways	64	3	2.76e-2	1.41e-1
Starch and sucrose metabolism (map00500)	KEGG pathways	64	3	3.24e-2	1.51e-1
Pentose and glucuronate interconversions (map00040)	KEGG pathways	64	3	3.76e-2	1.62e-1
Amino sugar and nucleotide sugar metabolism (map00520)	KEGG pathways	64	3	1.22e-1	3.37e-1
Biosynthasis of secondary metabolites (map0110)	KEGG pathways	64	17	2.76e-1	4.45e-1

Set: total number of selected metabolites; In set: the number of differential metabolites contained in this pathway.

### 3.6 Caffeine metabolism accumulation of multiple metabolites decreased with the deterioration of CKD

The caffeine metabolism pathway mapped by MBRole2.0 enriched 12 metabolites (Table 3), including Xanthine, Xanthosine, Theophylline, Theobromine, Caffeine, Paraxanthine, 7-Methylxanthine, 3-Methylxanthine, 1-Methylxanthine, 1,3,7-Trimethyluric acid, 5-Acetylamino-6-formylamino-3-methyluracil, and 5-Acetylamino-6-amino-3-methyluracil, which were circled in red in Figure 4A. The levels of paraxanthine, theobromine, caffeine, and theophylline decreased with renal deterioration (metabolites in red boxes in Figure 4A). Xanthosine and xanthine increased with renal deterioration (metabolites in green boxes in Figure 4A).

## 4 Discussion

In this study, we enrolled 145 CKD individuals who were divided into four groups based on mGFR. Many differential metabolites were screened out by metabolic profiling. Through pathway analysis of differential metabolites, we report for the first that the caffeine metabolism pathway is critical in CKD. We showed that in this pathway, paraxanthine, theobromine, caffeine, and theophylline decreased with poorer renal function, while xanthosine and xanthine increased. Caffeine was the most important metabolite, decreasing with deteriorating renal function.

Previous studies have found that coffee consumption may reduce the risk of CKD (Li et al., 2021), showing a negative association between caffeine intake and all-cause mortality in patients with CKD (Bigotte et al., 2019). However, other studies have shown that caffeine exacerbates hypertension in rats with polycystic kidney disease (Tanner and Tanner, 2001), potentiates the development of more severe tubulointerstitial changes, and increases focal glomerulosclerosis (Tofovic et al., 2002). High caffeine-sugar content increases the incidence of cardiovascular disease and tissue inflammation by altering lipid profiles and blood glucose (Eltahir et al., 2020). Yu *et al.* (Yu et al., 2016) found that acute caffeine intake causes an acute increase in blood pressure, while chronic caffeine intake decreases blood pressure; the latter may be related to a diuretic effect. Chronic caffeine consumption also reduces sodium absorption, contributing to its antihypertensive effects in salt-sensitive rats (Wei et al., 2018). This study found that caffeine metabolism was the most important pathway and that caffeine was the most important metabolite.

In our study, paraxanthine, theobromine, and theophylline decreased with poorer renal function. It is known that theobromine activates sirtuin one to reduce extracellular matrix accumulation in the kidneys of diabetic rats (Papadimitriou et al., 2015); a single dose of prophylactic theophylline has been shown to prevent acute kidney injury (AKI)/severe kidney dysfunction in term neonates with severe birth asphyxia (Bhatt et al., 2019). This study corroborated that theobromine and theophylline improve the progression of CKD. The effect of paraxanthine on CKD is still unclear. In this study, paraxanthine decreased with decreasing mGFR, which may also improve the progression of CKD.

We also found that xanthosine and xanthine increased with poorer renal function. Chen *et al.* (Chen et al., 2020) demonstrated that xanthosine is associated with significantly greater risks of CKD progression. Xanthine is an intermediate metabolite of uric acid (UA), converted by xanthine oxidase (XO). Previous research suggests a pathogenic role of hyperuricemia in the development of CKD (Uedono et al., 2015; Mallat et al., 2016). XO inhibitors have been suggested to slow the progression of kidney disease (Pisano et al., 2017) but this remains controversial (Kimura et al., 2018). The present study supports that xanthosine and xanthine are associated with CKD progression.

We found that metabolites that are associated with CKD progression can be converted to more ‘desirable’ metabolites, as shown in Figure 4A. This conversion involves many enzymes that are shown in Figure 4A. Thus, the regulation of these enzymes offers a potential target for CKD treatment. In subsequent research, we plan to focus on these enzymes. One limitation was that we did not record the baseline caffeine ingestion in our participants; the influence of caffeine ingestion on renal function in CKD should be observed in subsequent studies.

In this study, we found that in pyrimidine metabolism and histidine metabolism (Figures 4B,C), orotidine-5P, pseudouridine, 3-Ureidopropionate, urea, 1-Methyl-L-histidine, Hydantoin-5-propionate, N-Formimino-L-glutamate and imidazole lactate rise as mGFR decreases.​ Consistent with previously reported results, pseudouridine is extremely correlated with mGFR and might be an ideal biomarker for CKD (Peng et al., 2022). Urea is an endogenous marker in CKD (Weiner et al., 2015). The significance of the accumulation of the other six metabolites in CKD has not been reported. ​These eight metabolites have limited significance in the search for targets to protect kidney function. Moreover, in histidine metabolism, ergothioneine which is absorbed from the intestine through food intake may function as a major antioxidant (Cheah et al., 2017), and the protective effect on the kidney can be further investigated.

This study has potential limitations. Based on the metabolomic approach, we identified the significance of caffeine metabolism in CKD, but the mechanisms linking caffeine metabolism to CKD have yet to be clarified, and the exact intricate mechanism needs additional animal experiments and prospective studies. Future investigations will need to include more animal experiments and prospective studies to elucidate the mechanism and the significance of caffeine metabolism in CKD identified in this study.

## 5 Conclusion

In conclusion, metabolic profiling identified caffeine metabolism as the most important pathway in CKD progression. Decreased renal function was associated with decreased paraxanthine, theobromine, caffeine, and theophylline, and with increased xanthosine and xanthine. Caffeine was the most important metabolite associated with CKD deterioration.

## 6 Summary at a glance

Metabolic profiling was used to identify significant metabolic pathways in CKD progression. Four metabolic pathways were classified as important in the progression of CKD. Caffeine metabolism appears to be the most important pathway. Caffeine is the most important metabolite that decreases with the deterioration of CKD stage.

## Data Availability

The original contributions presented in the study are included in the article/supplementary material, further inquiries can be directed to the corresponding authors.
